# Community-acquired pneumonia: The need to broaden our diagnostic armamentarium

**DOI:** 10.7196/AJTCCM.2020.v26i1.063

**Published:** 2020-03-19

**Authors:** R Perumal, R N van Zyl-Smit

**Affiliations:** 1 Division of Pulmonology, Department of Medicine, Groote Schuur Hospital and University of Cape Town, South Africa; 2 Lung Clinical Research Unit, University of Cape Town Lung Institute, Cape Town, South Africa

## Editorial

Community-acquired pneumonia (CAP) is associated with significant mortality and morbidity, and high costs globally.^[1]^ The epidemiology of CAP demonstrates high regional and geographic variability with regard to age, risk factors, disease severity and causative pathogens. The identification of a pathogen in patients with CAP is an important, but seemingly elusive, goal. Nonetheless, clinicians should make every effort to identify a pathogen in hospitalised patients with CAP, as doing so will allow for targeted therapy. Narrow and directed therapy promotes antibiotic stewardship, which improves patient outcomes, minimises side-effects, shortens treatment duration, reduces the risk of developing complications such as *Clostridium difficile* infection and reduces the overall cost of care.

Despite the obvious benefits of individualised therapy, the failure to identify pathogens in the majority of patients has led to an empirical treatment strategy in most parts of the world. The choice of empirical therapy varies slightly between settings, but is generally based on local epidemiology and pathogen profile, local antibiograms, the risk-profiling of patients, travel and exposure histories and disease severity. This strategy has helped to standardise treatment approaches to some degree, promotes rational selection of antibiotics and has been demonstrated to improve clinical outcomes.^[2]^

The microbiological aetiology of CAP has been of much interest since the introduction of antibiotics for its treatment almost a century ago. In the pre-antibiotic era, *Streptococcus pneumoniae* caused 95% of the cases of pneumonia, but this has declined dramatically in the era of antibiotic therapy and pneumococcal vaccination. Globally, S. pneumoniae remains the most commonly identified cause of pneumonia, accounting for between 10 and 15% of hospitalised cases. However, with improved diagnostic capacity in recent years, additional pathogens have been identified, such as *Haemophilus influenzae, Staphylococcus aureus, Moraxella catarrhalis, Pseudomonas aeruginosa, Mycoplasma pneumoniae, Chlamydophila pneumoniae* and indeed, *Mycobacterium tuberculosis*. In a large population-based surveillance study of CAP in hospitalised adults, which employed systematic collection of urine, blood and respiratory specimens for culture, serological testing, antigen detection and molecular diagnostic testing, a pathogen was detected in only 38% of cases.^[3]^ Similarly to other rigorous studies of CAP aetiology, viral pathogens somewhat surprisingly accounted for twice as many cases as bacterial pathogens, and polymicrobial infection was not uncommon.^[4,5]^ As diagnostic capabilities improve further, we are likely to aim for a more individualised approach to the treatment of CAP. To do so, however, we must begin to characterise the local CAP epidemiology, including the profile of pathogens in our setting. Previous work by Nyamande *et al*. ^[4]^ revealed that systematic diagnostic investigations performed at a single centre in KwaZulu-Natal yielded a pathogen in over half the cases of hospitalised adults with CAP. Most interestingly, tuberculosis (TB) was the most commonly identified pathogen in both HIV-infected and non-infected patients in this setting of TB hyperendemicity. In a larger, multi-centre study,^[6]^ TB accounted for 28% of cases in hospitalised patients with CAP. Similarly to the emerging global picture, this South African (SA) study confirmed that viral pathogens were more common than bacterial pathogens, with rhinovirus being most commonly identified.^[3,5-7]^

In this issue of the *AJTCCM*, researchers from Tygerberg Hospital (TBH), one of two large tertiary hospitals in the Western Cape Province, reviewed their admissions for CAP to the intensive care unit (ICU) over a 12-month period.^[8]^ They found that patients with CAP accounted for 17.5% of the 423 admissions during that period, confirming the high burden of CAP in this setting.

However, although requiring ICU admission, the APACHE II score ranged from 6 to 39, with an overall ICU mortality rate of 21.6% – lower than the reported CAP mortality in ICUs in other settings.^[1-3,9]^ As with many studies of mortality in ICUs, this may very well represent local resource limitations, and varying thresholds for ICU admission. Nonetheless, the results are reassuring for a setting where the average age of patients with CAP is several decades lower than in most of the developed world, and in which pneumonia is a leading cause of death.

In keeping with published data from other parts of the country, TB was found to be a relatively common pathogen, accounting for roughly 22% of CAP admissions, representing the most common identifiable cause. This confirms the increasing presentation of TB as an acute pneumonia.^[10]^ Mazaza *et al*. ^[8]^ argue, and rightly so, that we should actively confirm or exclude TB in every patient admitted to the ICU. The location of their study, in the global epicentre of TB, reminds us that we should always seek to ‘know our epidemic, and know our response’ – the global rallying call for intensified local responses to global epidemics based on unique local disease epidemiology. Failing to identify TB as a cause of pneumonia in the ICU, conversely, may have disastrous consequences and may contribute to the ongoing high mortality of this curable infectious disease. The risks to ICU staff pursuant to TB transmission dynamics may be mitigated by the fact that most patients are managed on closed ventilation circuits. However, the implications for infection control remain significant. Adequate safety precautions, including, but not limited to, isolation of TB cases, must be available and carried out to prevent nosocomial transmission. And most importantly, an improved understanding of TB drug pharmacokinetics and optimised dosing in critical illness is desperately needed.

What is evident from this study is that the vast majority of cases of CAP admitted to an ICU had no pathogen identified. However, molecular tests for viral pathogens *M. pneumoniae* and *C. pneumoniae*, and urine testing for *S. pneumoniae* and *Legionella* spp., were not routinely performed. This may have significantly compromised the claim of a systematic evaluation for a causative pathogen in this cohort.^[5,7,11,12]^ In addition, the timing of sampling in relation to antibiotic exposure is not explicit, and is likely to have reduced the diagnostic yield of the performed tests. This is especially likely given the increasing pressure on emergency units to initiate antibiotics as soon as sepsis is suspected, which is usually prior to ICU admission.

Another interesting finding was the relatively common identification of highly susceptible *P. aeruginosa* in 12% of patients. No clear explanation for this occurrence is provided, but it is likely that *P. aeruginosa* is more ubiquitous in the community than we had thought. This low prevalence of phenotypic resistance was also recently reported by the sentinel surveillance laboratories of SA, and may help inform future empirical therapeutic decisions in patients with suspected *P. aeruginosa* infection.^[13]^

What is clear from this study is that if you do not test for a pathogen, you will not identify it. The ICU at TBH routinely tests all patients for TB using the GeneXpert test. The GeneXpert MTB/RIF assay, however, is not infallible, with an imperfect sensitivity and a clinically significant rate of false-positive results, especially in patients with previous TB. The authors do not present culture confirmation of the GeneXpert results – nor what percentage of GeneXpert-positive patients had prior TB, in order to confidently exclude false positive tests.

The findings of this study from a large SA hospital contribute to the arduous search for pathogens in our local burden of CAP. This information may inform empirical treatment choices, but needs to be expanded on by methodologically rigorous studies in this field. The challenge of developing a locally responsive empirical strategy is in balancing the risk of failure to treat, on the one hand, and the risks of overtreatment on the other.^[9]^ What is made clear by Mazaza *et al*. ^[8]^ is that unless we start looking, we may never know what we are up against, or how to tailor our response.
